# CRISPR/Cas9-mediated *PINK1* deletion leads to neurodegeneration in rhesus monkeys

**DOI:** 10.1038/s41422-019-0142-y

**Published:** 2019-02-15

**Authors:** Weili Yang, Yunbo Liu, Zhuchi Tu, Chong Xiao, Sen Yan, Xishan Ma, Xiangyu Guo, Xiusheng Chen, Peng Yin, Zhengyi Yang, Su Yang, Tianzi Jiang, Shihua Li, Chuan Qin, Xiao-Jiang Li

**Affiliations:** 1 Guangdong-Hongkong-Macau Institute of CNS Regeneration, Ministry of Education CNS Regeneration Collaborative Joint Laboratory, Jinan University, 510632 Guangzhou, Guangdong China; 2 Institute of Laboratory Animal Science, Chinese Academy of Medical Sciences and Peking Union Medical College, 100021 Beijing, China; 3 Department of Human Genetics, Emory University School of Medicine, Atlanta, GA 30322 USA; 4 Brainnetome Center, Institute of Automation, The Chinese Academy of Sciences, 100190 Beijing, China

**Keywords:** Developmental biology, Biological techniques

Dear Editor,

*PINK1* mutations cause autosomal recessive and early-onset Parkinson’s disease (PD) with selective neurodegeneration. Unfortunately, current *PINK1* knockout (KO) mouse^1,2^ and pig models^3,4^ are unable to recapitulate the selective and overt neurodegeneration seen in PD patients. Furthermore, endogenous Pink1 in the mouse brain is expressed at very low levels and can only be detected via immunoprecipitation,^5^ meaning that PINK1’s function in the mammalian brain needs to be assessed using larger animals that are closer to humans. We previously used CRISPR/Cas9 to target the monkey gene in one-cell stage embryos.^6^ Using the same approach, we designed two gRNAs to target exon 2 (T1) and exon 4 (T2), which encode a kinase domain in the *PINK1* gene of rhesus monkeys (Fig. 1a). CRISPR/Cas9 and PINK1 gRNAs were injected into one-cell stage rhesus monkey embryos. A T7E1 assay and sequencing of PCR products from the injected embryos showed high efficiency (61.5%) in targeting *PINK1* (Fig. 1b and Supplementary information, Fig. S1). Transfer of 87 embryos to 28 surrogate rhesus monkeys resulted in 11 pregnancies (39.2%) (Fig. 1b). Eleven fetuses developed to term and were born naturally. Of these live monkeys, eight carried *PINK1* mutations (M), and three were wild type (WT). However, three mutant monkeys (M1, M3 and M4) were newborn triplets that struggled to survive and died 3-4 days after birth. One WT newborn monkey also died after a difficult labor. Another mutant monkey (M2) died 7 days after birth without noticeable warning signs or symptoms. The other three mutant monkeys (M6, M7 and M8) have lived for three years; M5, however, reduced its food intake and showed weakness at the age of 1.5 years, and died 30 days after anesthesia for MRI examination.

**Fig. 1:**
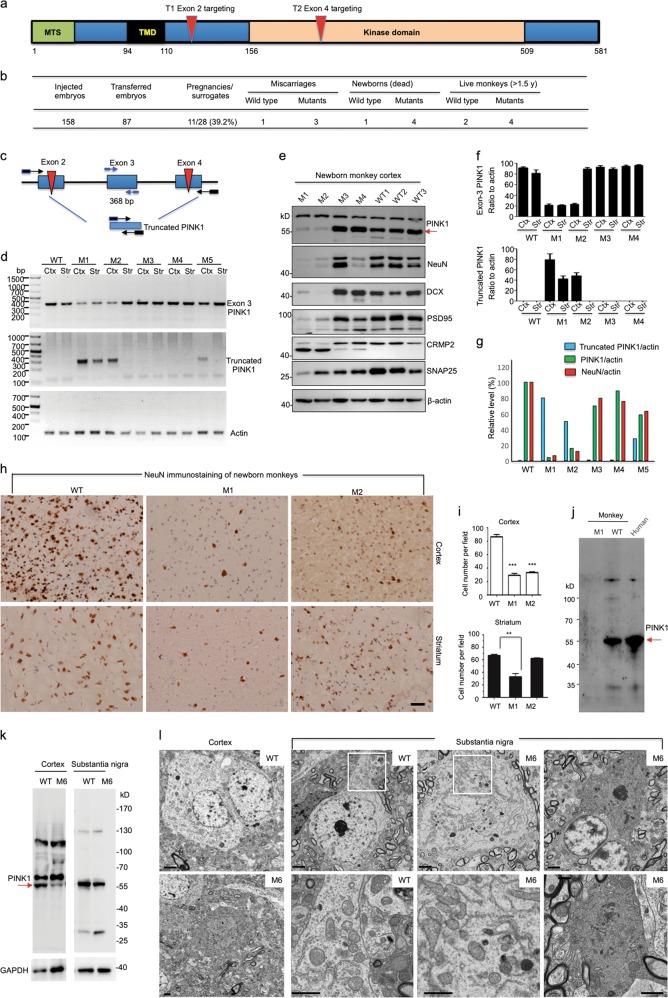
**a** PINK1 protein domains and targeted regions. MTS (mitochondrial targeting sequence), TMD (transmembrane domain), exon 2 (T2) and exon 4 (T4) targeting regions are indicated. **b** Summary of embryo injection, transfer, pregnancy, and newborn monkeys. **c** Diagram of PCR primers designed to determine the *PINK1* large deletion. **d** Large *PINK1* deletion in the cortex and striatum of M1, M2, M3, M4, and M5 monkeys. Exon 3 *PINK1* represents the remaining intact *PINK1*, whereas truncated PINK1 is generated by large deletion. **e** Western blot analysis of the brain cortical tissues of four *PINK1* mutant monkeys and three newborn wild-type (WT) monkeys. The tissues were probed with antibodies to PINK1, neuronal proteins (NeuN, PSD95, CRMP2, and SNAP25), β-actin, and doublecortin (DCX). **f** Quantitative analysis of the ratio of exon 3 *PINK1* or truncated *PINK1* product to actin revealed that the cortex (Ctx) and striatum (Str) of M1 and the cortex of M2 had an extensive large deletion. The results were obtained from three PCR experiments. **g** Inverse correlation of the rate of the large *PINK1* deletion (truncated PINK1/actin) with the relative levels of PINK1 (PINK1/actin) or NeuN (NeuN/actin) revealed by western blotting (*n* = 3 independent experiments via analysis of the cortical tissues in three WT and four mutant monkeys). **h** Representative immunostaining micrographs show loss of NeuN-positive neurons in the cortex of *PINK1* mutant monkeys (M1 and M2) compared with WT controls. Scale bar, 30 μm. **i** Quantitative assessment of NeuN-positive neurons in the cortex and striatum of newborn *PINK1* mutant monkeys (M1 and M2) and two WT control monkeys. **P < 0.05, ***P < 0.01. **j** Western blotting of lysates from WT or M1 monkey brain cortex and the human brain hippocampus. Red arrow indicates PINK1. **k** Western blot analysis of cortex and substantia nigra lysates of M6 monkey and age-matched 3-year-old WT monkey. Red arrow indicates PINK1. **l** Electron microscopy revealed degenerated cells in the cortex and substantia nigra of the M6 monkey. Degenerated neurons show electron-dense cytoplasm with no clear organelles and no identifiable nuclear membrane, and dark neurons are more obvious in the M6 substantia nigra. The enlarged micrographs beneath the corresponding WT and M6 images are from their boxed areas and show mitochondrial morphology. Scale bars, 2 μm

The mosaicism of CRISPR/Cas9-mediated mutations can induce different extents of PINK1 loss and phenotypes, allowing us to examine the relation of *PINK1* mutations with PINK1 expression and pathological changes. Indeed, a T7E1 assay and sequencing analysis of the targeted DNA regions revealed various types of DNA mutations (Supplementary information, Fig. S1). Importantly, we identified a large deletion (7,237 bp) between exon 2 and exon 4 in dead monkey tissues via PCR and sequencing of PCR products, as well as whole-genome sequencing (Fig. 1c and Supplementary information, Fig. S2). T7E1 analysis of several potential off-target genes in brain cortical tissues from *PINK1* mutant monkey (M1, M2, M3, and M4) and blood tissues from live *PINK1* mutant monkeys (M5, M6 and M7) revealed no mutations (Supplementary information, Fig. S3a). Whole-genome sequencing of M1, M2, and M3 monkeys showed no significant mutation rates in the top 20 potential off-target genes (Supplementary information, Fig. S3b) and analysis of 2,189 possible off-target sites with up to five mismatches of the gRNA sequences in the genome also revealed no off-targeting events (Supplementary information, Fig. S3c).

The large deletion in *PINK1* is different from point mutations found in humans and should completely eliminate PINK1 expression. To provide more evidence for the specific targeting of the *PINK1* gene and the resulting phenotype, we used western blotting to assess PINK1 expression and PCR to evaluate the relative degree of the large deletion by detecting the ratio of truncated DNA resulting from this deletion to the remaining intact exon 3 DNA (Fig. 1c–e). We found that ~65%–70% of *PINK1* alleles in M1 cortex and striatum and M2 cortex carry the ~7.2 kb deletion (Fig. 1f). Western blotting analysis of *PINK1* mutant monkey brains also confirmed differing extents of deficiency in PINK1, neuronal proteins (NeuN, PSD95, CRMP2, and SNAP25), and doublecortin (DCX) (Fig. 1e). All our results clearly showed that M1 and M2 monkey cortical tissues had the highest degree of the large *PINK1* deletion and the lowest level of PINK1 and neuronal proteins (Fig. 1g). Counting NeuN-positive cells also verified that M1 and M2 cortical and M1 striatal tissues had significantly fewer neuronal cells than WT controls (Fig. 1h, i). Moreover, we verified that PINK1 is also abundantly expressed in the human brain (Fig. 1j).

For the live monkeys, MRI and video monitoring studies revealed that 1.5-year-old adult monkeys with *PINK1* mutations showed significantly decreased gray matter density in the cortex (Supplementary information, Fig. S4a-c). M5 and M6 monkeys also displayed decreased movement despite no alteration in sleep behavior (Supplementary information, Fig. S4d, e, Movie S1). The M5 monkey lived up to 1.5 years and died 30 days after MRI examination. T7E1 assay revealed *PINK1* mutations in its brain and peripheral tissues (Supplementary information, Fig. S5a). Immunohistochemical studies showed reduced density of NeuN-positive neuronal cells and increased GFAP staining in the cortex and striatum of the M5 monkey compared with a 1.5-year-old WT monkey (Supplementary information, Fig. S5b). Because M5 monkey brain tissues were not isolated immediately after death for electron microscopic (EM) examination, we euthanized the symptomatic M6 monkey at the age of three years for EM to provide ultrastructural evidence for neurodegeneration. Analysis of M6 monkey brain genomic DNA also revealed the large deletion between *PINK1* exon 2 and exon 4 in various tissues (Supplementary information, Fig. S2b), and western blot analysis showed significantly decreased PINK1 expression in the cortex and substantia nigra compared with an age-matched WT monkey (3-year-old) (Fig. 1k). EM revealed degenerated neurons in the cortex, substantia nigra and striatum, as characterized by their electron-dense cytoplasm with no clear organelles and no identifiable nuclear membrane (Fig. 1l). Interestingly, in those degenerated neurons, the mitochondrial morphology is indistinguishable from WT monkey neurons.

The remarkable neuronal loss seen in *PINK1* mutant monkeys was not reported in *PINK1* KO mice^1,2^ or pigs,^3,4^ and may be associated with the primate-specific expression and function of PINK1. However, most patients with *PINK1* mutations do not show the same severe phenotypes as *PINK1* mutant monkeys. The differences in neurodegeneration and phenotypes between *PINK1* mutant monkeys and patients are very likely due to different types of *PINK1* mutations. Most *PINK1* mutations in humans are homozygous point mutations in one exon of the *PINK1* gene, with a few cases of heterozygous large deletions.^7–9^ The single locus mutations and heterozygous deletion may cause a partial loss of PINK1 expression or function and heterogeneity in PINK1 function, leading to different ages of onset with the earliest being 5 years,^7^ and various phenotypes in patients. On the other hand, the CRISPR/Cas9-mediated large deletion with other mutations can completely eliminate PINK1 expression and function, resulting in more severe phenotypes and neuropathology, as seen in some dead newborn monkeys (M1 and M2). It is possible that in humans, the complete loss of PINK1 leads to lethality during early development, therefore only those mutations causing a partial loss of PINK1 function are seen. Indeed, the mosaicism of CRISPR/Cas9-mediated mutations also led to live *PINK1* mutant monkeys that showed a partial loss of PINK1 and less severe neurodegeneration. Mounting evidence suggests that PINK1’s function is diverse^10^ and that its dysfunction is invovled not only in PD, but also in cancers and other diseases.^11,12^ Generation of *PINK1* mutant monkeys revealed the critical function of PINK1 in the primate brain and will provide a new tool to investigate the diverse functions of PINK1 and the pathogenesis related to PINK1 dysfunction.

## Supplementary information

Supplementary information

Supplementary movie S1

Supplementary movie S1 legend
