# Supplementary material for: CRISPR/Cas9-mediated PINK1 deletion leads to neurodegeneration in rhesus monkeys
Source: Cell Res. 2019 Feb 15;29(4):334–6. doi: 10.1038/s41422-019-0142-y (PMC6461954; doi:10.1038/s41422-019-0142-y)
Supplement: Supplementary file 1 — Supplementary information [file 41422_2019_142_MOESM1_ESM.pdf]

Supplementary information

Materials and Methods

Animals

Monkeys were housed by Chinese National standards, which are consistent with the standard outlined in the eighth edition of the NRC Guide for the Care and Use of Laboratory at the Institute of Laboratory Animal Science, Chinese Academy of Medical Sciences, Peking Union Medical College. This institution is fully accredited by the Association for Assessment and Accreditation of Laboratory Animal Care (AAALAC), International. The animal use and experiments followed the protocol that was approved by the Institutional Animal Care and Use Committee (IACUC) of the Institute of Laboratory Animal Science, Chinese Academy of Medical Sciences. This study occurred in strict compliance with the ‘‘Guide for the Care and Use of Laboratory Animals of the Institute of Laboratory Animal Science (est. 2006)’’ and ‘‘The use of non-human primates in research of the Institute of Laboratory Animal Science (est. 2006)’’ to ensure the safety of personnel and animal welfare.

Cas9/sgRNA vectors

Cas9 plasmid (MLM3613, Plasmid #42251) was used to express spCas9 nuclease (Streptococcus pyogenes) under the control of the CMV or T7 promoter. Dr. Liangxue Lai at The Guangzhou Institutes of Biomedicine and Health, Chinese Academy of Sciences provided p-T7-gRNA expression vectors, which were used for in vitro transcription of gRNAs. gRNAs were designed based on the targeted sequences in the monkey *PINK1* genes and were generated by inserting gRNAs into p-T7-gRNA via BbsI restriction sites. gRNA sequences are

as follows: *PINK1* exon 2 sgRNA: 5'-GGCTGGAGGAGTATCTGATAggg-3', *PINK1* exon 4 sgRNA: 5'-ccgGGTTCTCCGCGCTTTCACC-3'. Template DNAs for *in vitro* transcription were generated by PCR amplification of the gRNAs plasmids via the following primers: Sense (5'-GAAATTAATACGACTCACTATA-3'), Anti-sense (5'-AAAAAAAGCACCGACTCGGTGCCAC-3'). The PCR products were purified and transcribed *in vitro* by mMESSAGE mMACHINE T7 kit (Ambion, AM1344). Cas9 plasmids were linearized by PmeI and *in vitro* transcribed using MAXIscript T7 (Ambion, AM1312). The synthesized transcripts were added with poly-A using the *E. coli* Poly (A) Polymerase kit (NEB, M0276) and were purified using LiCl with an additional ethanol precipitation.

Ovarian stimulation and recovery of monkey oocytes

The methods for monkey ovarian stimulation and oocyte recovery are similar to those described in our previous studies (6). Regular cycling females arranging between 5-8 years of age were subjected to follicular stimulation using twice-daily intramuscular injections of 18 IU of recombinant human FSH (rhFSH) for 8 days, followed by 1000 IU of human chorionic gonadotropin (HCG) on day 9. Cumulus-oocyte complexes were isolated by surgery operation and aspiration 37 h post-rhCG. Follicular contents were placed in Hepes-buffered Tyrode's albumin lactate pyruvate medium (TALP-Hepes) containing 0.3% BSA at 37 °C, supplemented with 5 IU/mL of heparin (Sigma, Inc.). Oocytes were stripped of cumulus cells with pipetting for 45-60 s, then filtered through a 70 µm cell strainer, and collected in a 60 mm petri dish containing 5-7 mL of TALP-Hepes. Oocytes were collected under a dissecting microscope to separate GV (intact germinal vesicle), metaphase I (GVB, no germinal vesicle, no polar body), metaphase II (MII, first polar body present) from other dead oocytes. Oocytes

were rinsed and then transferred to 50 μL pre-equilibrated maturation medium containing Connaught Medical Research Laboratories medium 1066 (CMRL-1066; Invitrogen Inc.) supplemented with 10% heat-inactivated fetal bovine serum (FBS), 40 μg/mL sodium pyruvate, 150 μg/mL glutamine, and 550 μg/mL calcium lactate under mineral oil. Immature oocytes such as GVB or GV cells were cultured in a 50 μl TALP-Hepes under 6% CO_2 at 37.5 °C for up to 24 h.

Male macaques were electro-ejaculated with a current isolation stimulator (JL-C4 V2a, JIALONG, China) equipped with electrocardiographic pad electrodes for direct penile stimulation (30-50 V, 20-ms duration, 18 pulses/s). Semen samples were collected into 15-mL tubes. Ejaculated sperm were diluted to 2×10^5 in 10% polyvinylpyrrolidone (PVP) to reduce motility and placed in a separate drop on the manipulation dish. A single sperm aspirated from the sperm drop into the injection needle, was transferred to the oocytes in the TALP-Hepes drop. MII oocytes immobilized with a holding pipet on the polar body at the 6 o'clock or 12 o'clock position, and then injected with a sperm through a needle through the zona into the cytoplasm (ICSI). After ICSI, oocyte was washed twice in Hamster Embryo Culture Medium 9 (HECM-9) before being transferred into a pre-equilibrated 50 μL drop of HECM-9, covered with mineral oil and incubated at 37.5 °C with 6% CO_2 for 8-10 h. Oocytes with a second polar body and two pronuclei arising after ICSI were confirmed successful fertilization. Zygotes were injected with Cas9 mRNA (200 ng/L) and gRNAs (50 ng/L), and the injected zygotes were cultured for embryo development. Embryos at 4-8 cell stages were used for transfer or divided into single blastomere for PCR. The pronuclear formation was recorded 16-20 h post-ICSI, and the progression of embryo growth was recorded daily.

Cas9/sgRNA injection of one-cell embryos

The zygotes were injected with Cas9 mRNA (200 ng/L) and gRNAs (50 ng/L each). Microinjections were performed in the cytoplasm of zygotes using a Narishige (Narishige Inc. Japan) microinjection system under standard conditions. The zygotes were cultured in embryo culture medium-9 (HECM-9) containing 15% fetal calf serum (Hyclone Laboratories, SH30088.02) at 37.5 °C in 6% CO_2. Cleaved embryos of high quality at the 4-cell stage were transferred into the oviduct of the matched recipient female monkeys. Typically, three embryos were transferred into each female. Ultrasonography detected pregnancies 30-35 days after the embryo transfer. Both clinical pregnancy and number of fetuses were confirmed by fetal cardiac activity and presence of a yolk sac as detected by ultrasonography.

PCR analysis of targeted monkeys and T7E1 Assay

The genomic DNAs were isolated from monkey tissues. The PINK1 DNAs including the target sites were amplified by PCR with primers for each site as follows: PINK1 exon 2 (forward: 5'-ccaggctgagcagtagaa-3', reverse: 5'-tgaacctaatccctgggtga-3'. PINK1 exon 4 (forward, 5'-ccaggctgagcagtagaa-3', reverse: 5'-tgaacctaatccctgggtga-3'). PINK1 primers for large deletion determination (forward: 5'-tacccagaaaagcaagccagggcct-3', reverse: 5'-acttcttcataacgaggaaca-3'). PINK1 exon 3 primer (forward: 5'-acacaatgagccaggagctg-3', reverse: 5'-cagagggcactgacctgtaa-3'). The PCR was performed by initial incubation at 95 °C for 5 min, followed by 35 cycles at 95 °C for 30 s, 62 °C for 45 s, and 72 °C for 45 s. T7E1 assay was used to analyze the PCR products to detect the targeted DNA mutations. PCR products corresponding to genomic modifications were then subcloned into a TA-cloning vector for sequencing to verify the mutation sequences.

Western blot analysis, immunohistochemistry and electron microscopy

Human brain hippocampus tissue was obtained with informed consent under a protocol approved by The First Affiliated Hospital of Jinan University Institutional Review Board. Adult human brain hippocampal specimens involved in epileptic foci were obtained from surgeries for treating epilepsy. Tissue was immediately frozen in liquid nitrogen after resection. Human or monkey brain tissues were lysed in ice-cold RIPA buffer (50 mM Tris, pH 8.0, 150 mM NaCl, 1 mM EDTA, pH 8.0, 1 mM EGTA, pH 8.0, 2% SDS, 0.5% DOC, 50 mM NaF and 1% Triton X-100) containing Halt protease inhibitor cocktail (Thermo Scientific) and PMSF. The lysates were incubated on ice for 30 min, sonicated, and centrifuged at the maximum speed for 10 min. Equal amounts of proteins from the supernatants determined by BCA assay were resolved by SDS-PAGE and subjected to western blot analysis with appropriate primary antibodies: anti-NeuN (Millipore, MAB377), anti-GFAP (DAKO), anti-GFAP (Thermo), anti-DCX (CST, #4604), anti-SNAP25 (CST, #5308), anti-CRMP2 (CST, #9393), anti-PSD95 (CST, #3409), anti-PINK1 (Novus Biological, BC100-49), anti-β-actin (Santa Cruz, 47778), and anti-GAPDH (Gene Tex, GTX100118). Acquired images were subjected to densitometric quantitation using ImageJ software.

For immunohistochemistry, monkey brain tissues were fixed overnight (12-16 h) in 4% paraformaldehyde in 0.01 M PBS, and then transferred into 30% sucrose at 4 °C to let the brain completely sink to the bottom of the tube. Brain tissue was sectioned at 20 μm using a cryostat at −19 °C. Monkey tissue slides were fixed for 10 min in 4% paraformaldehyde in 0.01 M PBS at room temperature, blocked with 0.1% Triton X-100/2% NGS/3% BSA/1× PBS for 30 min, and incubated with primary antibodies to relative proteins in 3% BSA/2% NGS/1× PBS overnight at 4 °C. The slices were washed three times with 1× PBS and rinsed with secondary antibodies.

For electron microscopy (EM), M6 and age-matched (3 year old) monkeys were deeply anesthetized by intraperitoneal injection of 0.3-0.5 mL of atropine, followed by 10-12 mg of ketamine and 15-20 mg of pelltobarbitalum natricum per kg body weight. The freshly isolated brain tissues from sacrificed M6 and age-matched (3 year old) monkeys were fixed with 2.5% glutaraldehyde/0.1 M PB overnight at 4 °C. Brains were sectioned into 50 μm using a vibratome (Leica, VT1000s) and the sections were processed for electron microscopic examination. In brief, all sections were osmicated in 1% OsO_4 in 0.1 M PB and embedded in Eponate12 (Ted Pella). The dried brain sections were cut into ultrathin sections (60 nm) with a Leica Ultracut S ultramicrotome under a Hitachi H-7500 transmission electron microscope equipped with a Gatan Bio-Scan CCD camera at Emory University.

Off-target analysis and whole genome sequencing analysis

Potential off-target sites (OTs) of sgRNA guided Cas9 endonucleases for the *PINK1* gene were predicted by a bioinformation-based search tool (http://www.rgenome.net/cas-offinder/). Whole-genome sequencing were performed to analyze the off-target loci. Top 20 off-target loci in the gRNA targeted sequences were selected by blasting the *PINK1* gRNAs sequences in the monkey genome for analyzing and comparing with *PINK1* targeting rates, which were determined by CRISPR/Cas9 targeted and non-targeted monkeys.

Novogene prepared the DNA library preparation and conducted whole genome sequencing on the monkey genomic DNA. The edited monkey genome (*Macaca mulatta*) was used to produce a custom-made index file, and PEMapper was then used to map sequencing data to the custom-made index file. For each on-target and off-target locus, 1 kb of flanking region to each side of the locus

was added. We next used the pileup file generated by PEMapper to retrieve the base pair-level sequencing read coverage and reported the average.

For large deletion test, short reads were aligned using the Burrows-Wheeler Aligner (BWA) with default parameters. SAMtools was used to convert between SAM and BAM file format, and Picard tools to sort alignments. Sorted BAM files were uploaded to UCSC genome browser (http://genome.ucsc.edu) for visualization and analysis.

Genomic sites with sequence similarity to the T1 and T2 gRNAs were identified by a base-by-base scan of the entire monkey genome, allowing for ungapped alignments with up to 5 mismatches. Off-target sites juxtaposed to an ‘NGG’ PAM site were identified by comparing with the genomic DNA sequences of wild-type rhesus monkeys from University of Nebraska Nonhuman Primate Genome Center (http://www.unmc.edu/rhesusgenechip/index.htm.) and Ensemble (Macaca mulatta *8.0.1 edition).

Monkey behavioral studies

Monkey behavioral studies were conducted at the Institute of Laboratory Animal Science, Chinese Academy of Medical Sciences. Four *PINK1* mutant monkeys (1.5 years of age) and four age-matched control monkeys were examined. Monkeys were individually observed in an observation cage (1.4 × 0.9 × 1.15 m) that was similar to their home cage. All movement activities were video-record without interruption for 30 min each day for 6 consecutive days. For sleep examination, monkeys were video recorded from 8 pm to 6 am. The wake-up times and duration of each wake-up were measured by a Vigie Primates image analyzer system (View point, Lyon, France, Version: 4.7.0.520). Three experiments were performed on each monkey, and each experiment monitored the sleep behavior for five consecutive days.

Statistical analysis

Two-tailed Student's *t*-test (unpaired) was used to compare differences between age-matched control and mutant monkeys. Statistical analyses were performed with Excel. Values are represented in the text as mean ± SEM.

Figure S1

a

Code	Gender	Mutation	Age
M1	M	T1 and T2	Postnatal (166 d)
M2	F	T1 and T2	Postnatal (188 d)
M3	M	T1 and T2	Postnatal (166 d)
M4	M	T1 and T2	Postnatal (166 d)
M5	M	T1 and T2	Dead (1.5 yr)
M6	F	T1 and T2	Alive (3 yr)
M7	F	T1	Alive (3 yr)
M8	M	T1	Alive (3 yr)

b

Mutation analysis of monkey cortex of M1 to M8 by T7EN1 cleavage assay

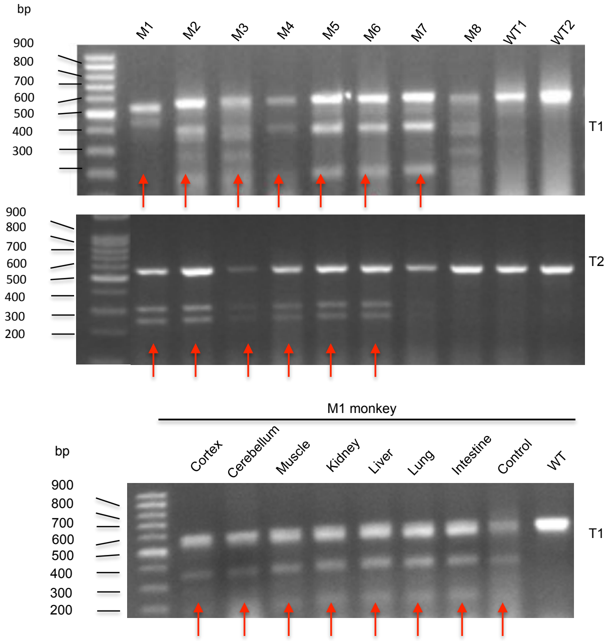

c

Exon-2 R L E E Y L I G Q S I G

T1

WT CGG CTG GAG GAG TAT CTG ATA GGG CAG TCC ATT GGC

Mut-1 CGG CTG GAG GAG TAT CTG G-A GGG CAG TCC ATT G----- (-40bp)

Mut-2 CGG CTG GAG GAG --- --- --- ---------------G –AG TCC ATT GGC (-12bp)

Mut-3 CGG CTG GAG GAG --- --- ------------------G –AG TCC ATT GGC (-12bp)

Mut-4 CGG CTG GAG GAG -----------GA- --- ---GCAG TCC ATT GGC (-9bp; +2bp)
CA

Mut-5 CGG CTG GAG GA- --- --- --A GGG CAG TCC ATT (-9bp)

Mut-6 CGG CTG GAG GAG -----------GA- --- ---GCAG TCC ATT GGC (-9bp; + 2bp)

Mut-7 CGG CTG GAG GA-------- --- --- --A GGG CAG TCC ATT GGC AAG (-9bp)

Mut-8 CGG CTG GAG GAG TAC CTG ATA GGG CAG TCC
CGG CTG GGG GAG TAT CTG ATA GGG CAG TCC (Point mutation: A-G)

Exon4 I I R V L R A F T S S V P

T2

WT ATC ATC CGG GTT CTC CGC GCT TTC ACC TCT TCC GTG CCG

Mut-1 ATC ATC CGG ------------------------T TTC ACC TCT TCC GTG CCG (-11bp)

ACC
Mut-2 ATC ATC CG---------------------------CT TTC ACC TCT TCCGTG CCG (-11;+3bp)
ATC ATC ---GG GTT CTC CGC GCT TTC ACC TCT TCC GTG CCG (-1bp)

Mut-3 AT------------------------CTC CGC GCT TTC ACC---------CC ACG CCT (-14bp)

Mut-4 ATC A----------------T CTC CGC GCT TTC ACC T-------CC GTG CCG (-10bp,-7bp)

Mut-5 ATC CGG GTT CTC CΔC GCT TTC ACC +1bp; point mutation(G-A)
G

Mut-6 ATC ATC CGG GTT CTC CGC GCT TTC ACC T-------CC GTG CCG (-7bp)

Supplementary information, Fig. S1. Generation of *PINK1* mutant monkeys via CRISPR/Cas9. a Summary of newborn and live monkeys that were targeted by CRISPR/Cas9. The age of death or survival is in parenthesis. Blood samples were used for genotyping the live monkeys, while brain cortex tissues were used for genotyping the dead monkeys (red). **b** T7E1 digestion of T1 and T2 target site DNAs from the cortex tissue of PINK1 mutant monkeys (upper panel) and T1 target site of various M1 monkey tissues (lower panel) showed *PINK1* mutations (red arrows). WT: wild type. **c** DNA sequencing of M1-M8 monkeys (M1-M6 DNA from brain tissues; M7-M8 DNA from blood tissues) showed various mutations in *PINK1* T1 and T2 target sites.

Figure S2

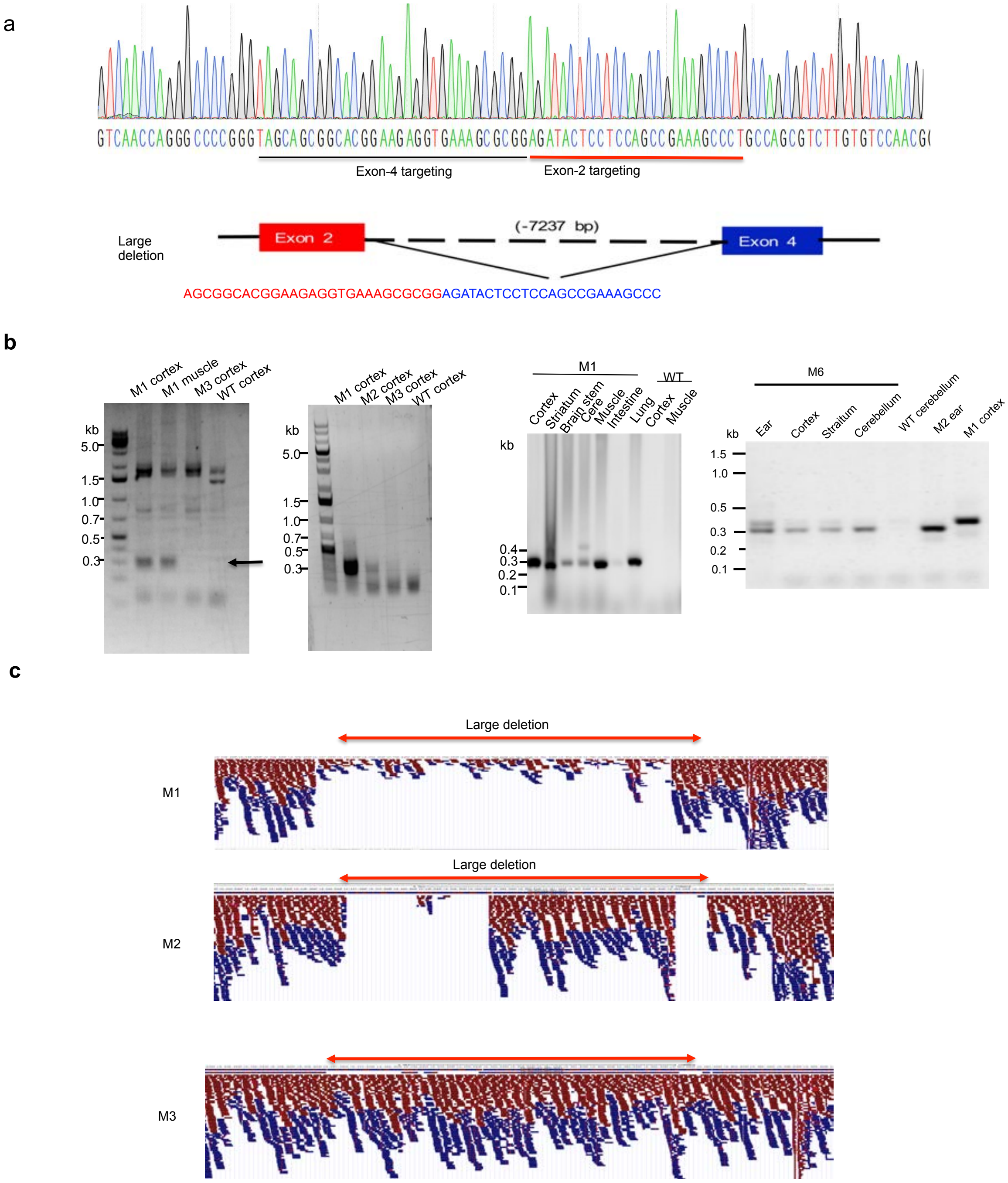

a
GTCAACCAGGGCCCCGGGTAGCAGCGGCACGGAAGAGGTGAAAGCGCGGAGATACTCCTCCAGCCGAAAGCCCTGCCAGCGTCTTGTGTCCAACG
Exon-4 targeting
Exon-2 targeting
Large deletion
Exon 2
(-7237 bp)
Exon 4
AGCGGCACGGAAGAGGTGAAAGCGCGGAGATACTCCTCCAGCCGAAAGCCC
b
M1 cortex
M1 muscle
M3 cortex
WT cortex
kb
5.0
1.5
1.0
0.7
0.5
0.3
M1 cortex
M2 cortex
M3 cortex
WT cortex
M1
WT
Cortex
Striatum
Brain stem
Cere
Muscle
Intestine
Lung
Cortex
Muscle
0.4
0.2
0.1
M6
Ear
Cortex
Straitum
Cerebellum
WT cerebellum
M2 ear
M1 cortex
c
Large deletion
M1
M2
M3

Supplementary information, Fig. S2. A large *PINK1* gene deletion in *PINK1* mutant monkeys. **a** DNA sequencing result revealed a large deletion of 7,237 bp between exon 2 and exon 4 in the monkey *PINK1* gene. **b** M1, M2 and M6 had the large deletion, which was revealed by PCR analysis of *PINK1* in their tissues. **c** Whole genome sequence data from the brain tissues showed M1 and M2 had large deletion between PINK1 exon 2 (T1) and exon 4 (T2) as compared with M3.

Figure S3

a

b

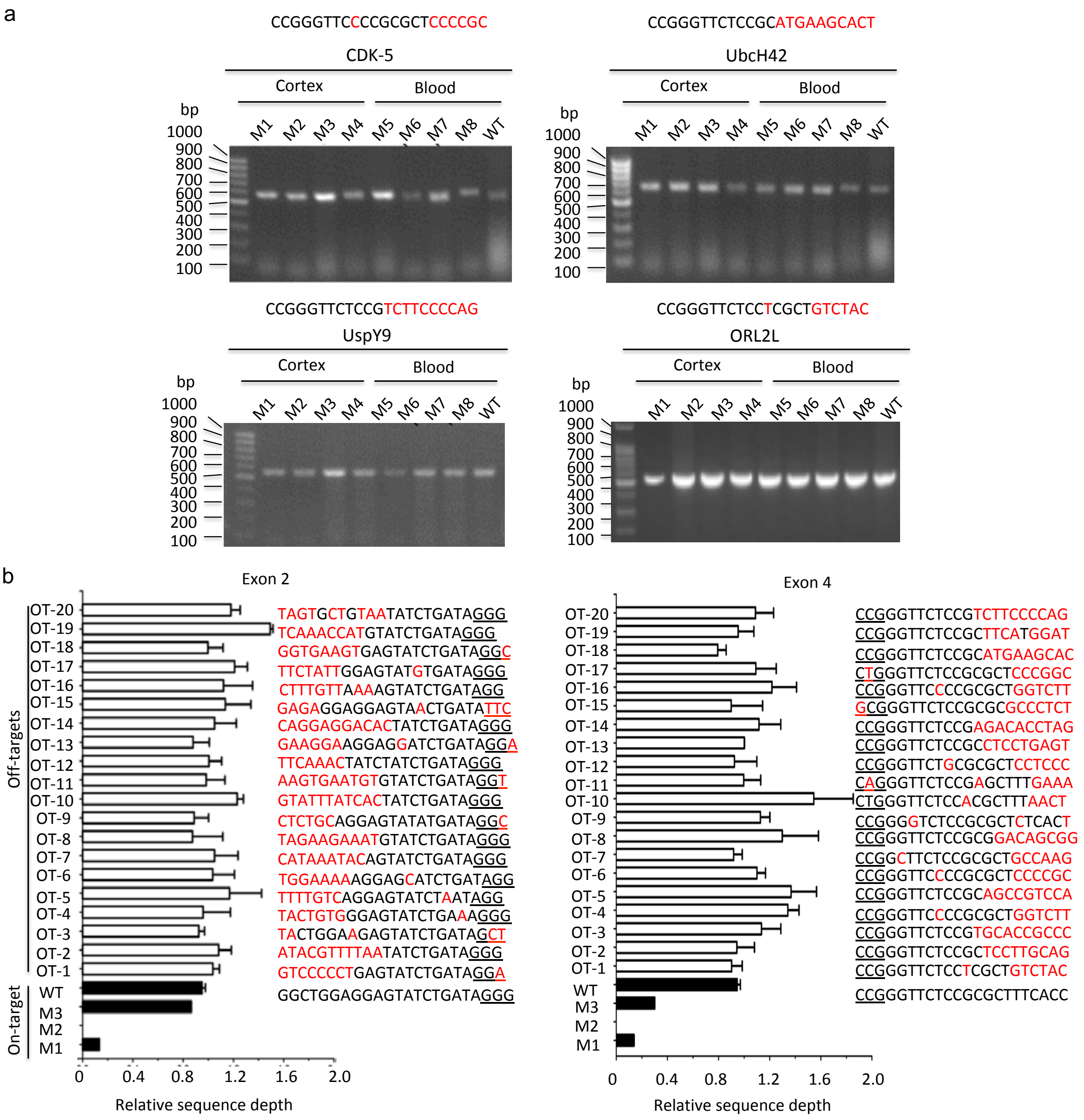

c

Summary of variants detected by whole-genome sequencing

			M1	M2	M3
	Mismatch	NGG genomic site	No. of off-targets	No. of off-targets	No. of off-targets
T1	0	0	0	0	0
	1	0	0	0	0
	2	0	0	0	0
	3	7	0	0	0
	4	57	0	0	0
	5	583	0	0	0
T2	0	0	0	0	0
	1	0	0	0	0
	2	1	0	0	0
	3	11	0	0	0
	4	120	0	0	0
	5	1410	0	0	0

Supplementary information, Fig. S3. Off-target assay and whole-genome sequencing. a T7E1 digestion analysis of potential off-target genes (CDK15, UbcH42, UspY9 and OR2L2) isolated from the brain cortical tissues of dead monkeys (M1, M2, M3, M4, M5, M6) and blood samples from live monkeys (M7, M8) as well as a wild-type control. No mutation events were found. Mismatched nucleotides are indicated in red. **b** Whole-genome sequencing analysis showing rare off-target mutations in the cortex of M1, M2, and M3. Genomic DNAs from the cortical tissues of *PINK1* mutant monkeys were subjected to whole-genome sequencing. Relative sequencing depth for the *PINK1* T1 and T2 targeted by CRISPR/Cas9 and 20 most likely off-target loci was calculated by normalizing the number of mapped reads in those loci to the genome-wide average of mapped reads. The data are presented as means ± SE (n = 3). For on-target analysis of *PINK1* mutant monkeys (M1, M2, M3), the mapped DNA read rates on the targeted region were obtained by comparing with untargeted sequence reads. Mismatched nucleotides are indicated in red. The rates for WT were obtained from whole-genome sequences of wild-type rhesus monkeys (n = 4). Mutations caused by Cas9 cutting led to a reduced number of mapped reads, thereby a reduced relative sequencing depth. **c** Summary of variants detected by whole-genome sequencing in PINK1 mutant monkey (M1, M2, and M3) cortex tissues. No off-target was detected in any known genes or exons.

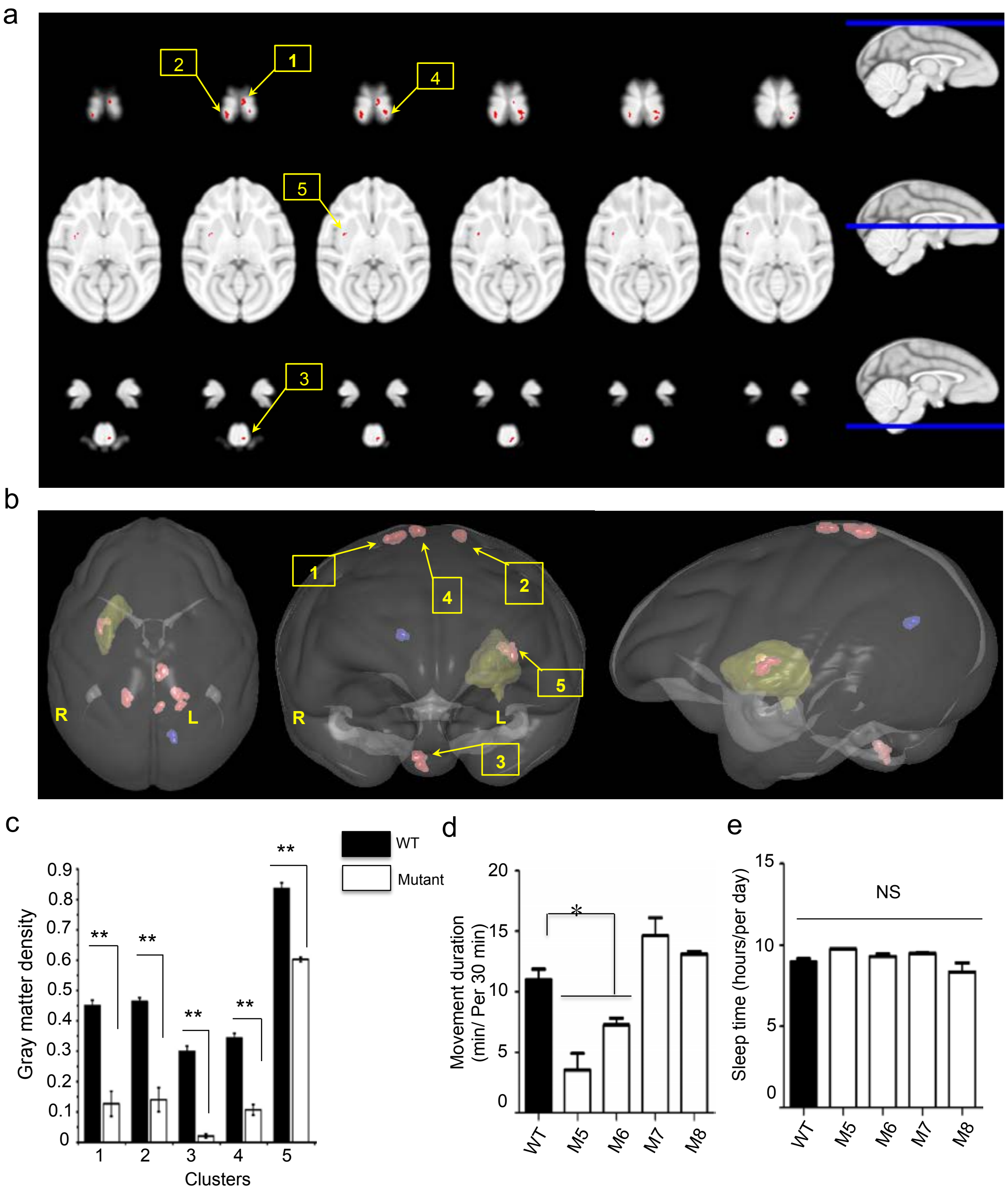
a
2
1
4
5
3
b
1
4
2
5
3
R
L
R
L
c
WT
Mutant
Gray matter density
0.9
0.8
0.7
0.6
0.5
0.4
0.3
0.2
0.1
0
**
1
2
3
4
5
Clusters
d
Movement duration (min/ Per 30 min)
20
15
10
5
0
*
WT
M5
M6
M7
M8
e
Sleep time (hours/per day)
15
10
5
0
NS
WT
M5
M6
M7
M8

Supplementary information, Fig. S4. MRI imaging data and movement activity of live *PINK1* mutant monkeys. a MRI shows reduced gray matter density in live *PINK1* mutant monkeys. In each row, 5 consecutive axial slices are shown with slice thickness of 0.3 mm, and the rightmost sagittal image indicates the position of those slices. Five brain regions indicated by numbers in yellow boxes represent different areas in the gray matter. **b** Based on 2D imaging data in **a**, 3D images consisting of clusters in a glass brain was constituted to show the alteration in the density of gray matter. Cluster 1: the right parietal cortex, Cluster 2: the left parietal cortex, Cluster 3: the right pons and medulla, Cluster 4: the right parietal cortex, Cluster 5: the left putamen. **c** Quantitative summary of the reduced gray matter density in *PINK1* mutant monkey brains. The gray matter density is decreased in distinct brain regions in *PINK1* mutant monkeys at the age of 1.5 year (n = 4 for *PINK1* mutant monkeys (M5, M6, M7, M8) and n = 4 for age-matched WT controls). Two of them are in the right parietal cortex, 1 in the left parietal cortex, 1 in the left putamen, and 1 in the right pons and medulla. No significant difference was identified in the intracranial volume, tissue-specific volumes, or regional volumes between mutant (M) and WT. The values (mean ± SE) from distinct brain regions (clusters 1-5) were shown. **$P < 0.01$. Statistical differences between groups were determined by Student's t-test. **d, e** Movement activities (**d**) and sleep time (**e**) of live *PINK1* mutant monkeys and age-matched wild-type control animals at 1.5 years of age. Each monkey was examined 6 times for consecutive 6 days, and the average values (mean ± SE) were presented. When compared with the WT controls, M5 and M6 showed reduced distance and duration in movement. The WT group (n = 4) was used to compare with each individual *PINK1* mutant monkey using Student's t-test. *$P = 0.0318$. NS: not significant.

Figure S5

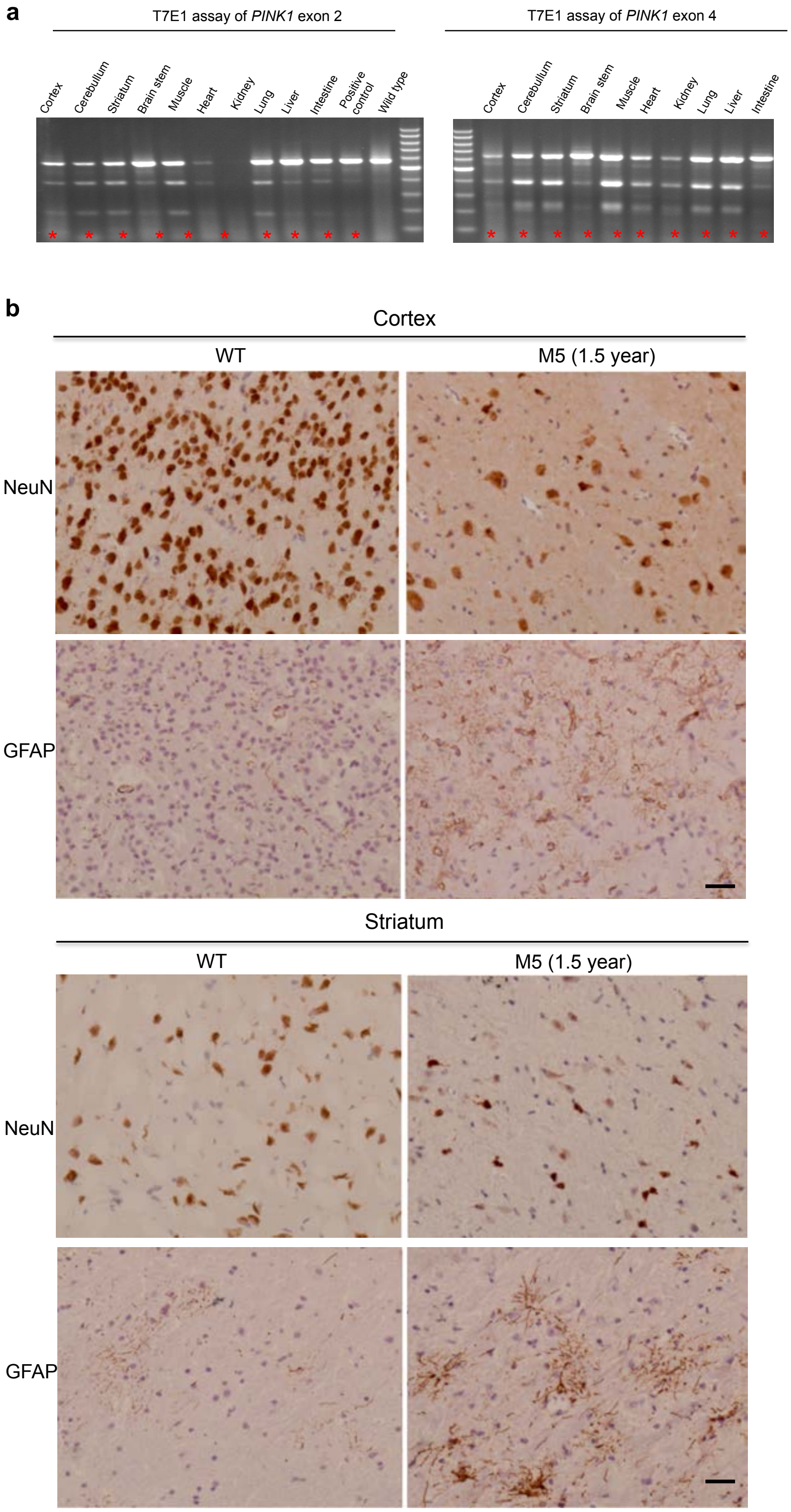
a
T7E1 assay of PINK1 exon 2
Cortex
Cerebullum
Striatum
Brain stem
Muscle
Heart
Kidney
Lung
Liver
Intestine
Positive control
Wild type
T7E1 assay of PINK1 exon 4
Cortex
Cerebullum
Striatum
Brain stem
Muscle
Heart
Kidney
Lung
Liver
Intestine
b
Cortex
WT
M5 (1.5 year)
NeuN
GFAP
Striatum
WT
M5 (1.5 year)
NeuN
GFAP

Supplementary information, Fig. S5. Neurodegeneration in adult PINK1 mutant monkey (M5). a T7E1 digestion of the targeted *PINK1* T1 and T2 in different tissues from 1.5-year old monkey (M5). Red asterisk indicates mutant DNAs. **b** Immunohistochemical Analysis of M5 and wild-type monkey brains. NeuN or GFAP immunostaining of the cortex and striatum of 1.5-year-old M5 and wild-type monkeys. Scale bars, 50 μm.
