# Supplementary material for: CRISPR/Cas9-mediated PINK1 deletion leads to neurodegeneration in rhesus monkeys
Source: Cell Res. 2019 Feb 15;29(4):334–6. doi: 10.1038/s41422-019-0142-y (PMC6461954; doi:10.1038/s41422-019-0142-y)
Supplement: Supplementary file 3 — Supplementary movie S1 legend [file 41422_2019_142_MOESM3_ESM.pdf]

Supplementary information, Movie S1

Representative videos of M6 (left) and an age-matched wild-type monkey (right). The videos were recorded at the age of 1.5 years.
